# Comparison of structural variant callers for massive whole-genome sequence data

**DOI:** 10.1186/s12864-024-10239-9

**Published:** 2024-03-28

**Authors:** Soobok Joe, Jong-Lyul Park, Jun Kim, Sangok Kim, Ji-Hwan Park, Min-Kyung Yeo, Dongyoon Lee, Jin Ok Yang, Seon-Young Kim

**Affiliations:** 1 Korea Bioinformation Center (KOBIC), Korea Research Institute of Bioscience and Biotechnology (KRIBB), Daejeon, 34141 Republic of Korea; 2 Aging Convergence Research Center, Korea Research Institute of Bioscience and Biotechnology (KRIBB), Daejeon, 34141 Republic of Korea; 3 Department of Convergent Bioscience and Informatics, College of Bioscience and Biotechnology, Chungnam National University, Daejeon, 34134 Republic of Korea; 4 Department of Bioscience, University of Science and Technology (UST), Daejeon, 34113 Republic of Korea; 5 Department of Pathology, Chungnam National University School of Medicine, Daejeon, 35015 Republic of Korea; 6 Department of Bio and Brain Engineering, Korea Advanced Institute of Science and Technology (KAIST), Daejeon, 34141 Republic of Korea; 7 Department of Functional Genomics, University of Science and Technology (UST), 34113 Daejeon, Republic of Korea

**Keywords:** Large-scale genomic dataset, Structural variation, Whole-genome sequencing

## Abstract

**Background:**

Detecting structural variations (SVs) at the population level using next-generation sequencing (NGS) requires substantial computational resources and processing time. Here, we compared the performances of 11 SV callers: Delly, Manta, GridSS, Wham, Sniffles, Lumpy, SvABA, Canvas, CNVnator, MELT, and INSurVeyor. These SV callers have been recently published and have been widely employed for processing massive whole-genome sequencing datasets. We evaluated the accuracy, sequence depth, running time, and memory usage of the SV callers.

**Results:**

Notably, several callers exhibited better calling performance for deletions than for duplications, inversions, and insertions. Among the SV callers, Manta identified deletion SVs with better performance and efficient computing resources, and both Manta and MELT demonstrated relatively good precision regarding calling insertions. We confirmed that the copy number variation callers, Canvas and CNVnator, exhibited better performance in identifying long duplications as they employ the read-depth approach. Finally, we also verified the genotypes inferred from each SV caller using a phased long-read assembly dataset, and Manta showed the highest concordance in terms of the deletions and insertions.

**Conclusions:**

Our findings provide a comprehensive understanding of the accuracy and computational efficiency of SV callers, thereby facilitating integrative analysis of SV profiles in diverse large-scale genomic datasets.

**Supplementary Information:**

The online version contains supplementary material available at 10.1186/s12864-024-10239-9.

## Background

With the boom in large-scale high-throughput sequencing data, the analyses of numerous sequences, including variants and structural variations (SVs), in various population and disease studies have increased exponentially [1–4]. Additionally, in contrast to the array comparative genomic hybridization approach, next-generation sequencing (NGS) technology enables simultaneous detection of all variants [5]. Moreover, it allows breakpoint identification for subsequent analyses, such as through inferring mutation mechanisms. Furthermore, the sequencing-based approach will enable researchers to derive information regarding SVs from short- and long-read sequencing for predicting potential disease risk. Although population-scale studies of SVs are time-consuming and require high-performance computing resources, they are becoming more common worldwide due to the development of long-read sequencing technology.

SVs are generally defined as the variations between individuals of the same species and can include genomic deletions, insertions, duplications, inversions, and translocations (≥ 50 bp). SVs, unlike small-sized variants, are the most significant cause of genetic variation among individuals. SVs can cause genetic diversity, phenotypic variation, and various diseases [6]. They are approximately ≥ 1 kb, and SVs > 1 kb have a more immediate and marked impact [7]. Another consideration is the role of copy number variations (CNVs), which indicate imbalanced deletions and duplications, which are common in normal populations [8–11]. Notably, CNVs partially explain the differences in various traits in a non-disease population and play a key role in genetic evolution and mutation accumulation [12, 13].

With the increasing importance of SV studies worldwide, several SV analysis tools have been developed [14, 15]. Although these methods are suitable for analyzing vast amounts of genomic data in large-scale population studies, researchers require reliable information on the performance of these tools and the necessary computational infrastructure, respectively. Existing SV benchmarking studies focused on the accuracy of SV callers or used reference SVs of limited samples [6]. NA12878 has been established as the standard reference set for germline SVs and is frequently employed as a benchmark. Nevertheless, due to the dearth of reference SVs representing a diverse array of ethnic groups, including Asians, this approach may produce biased outcomes. Therefore, it is essential to conduct further comparative analyses of SV callers with a range of datasets that feature samples from different populations, along with simulated datasets [16], to ascertain the effectiveness of large-scale SV calling procedures and computing capabilities. Moreover, it is necessary to compare various read-depth NGS data [17–19] and verify the efficiency of large-scale SV calling according to computing power. Comparing the validity of the results for genotype prediction is also necessary.

**Fig. 1:**
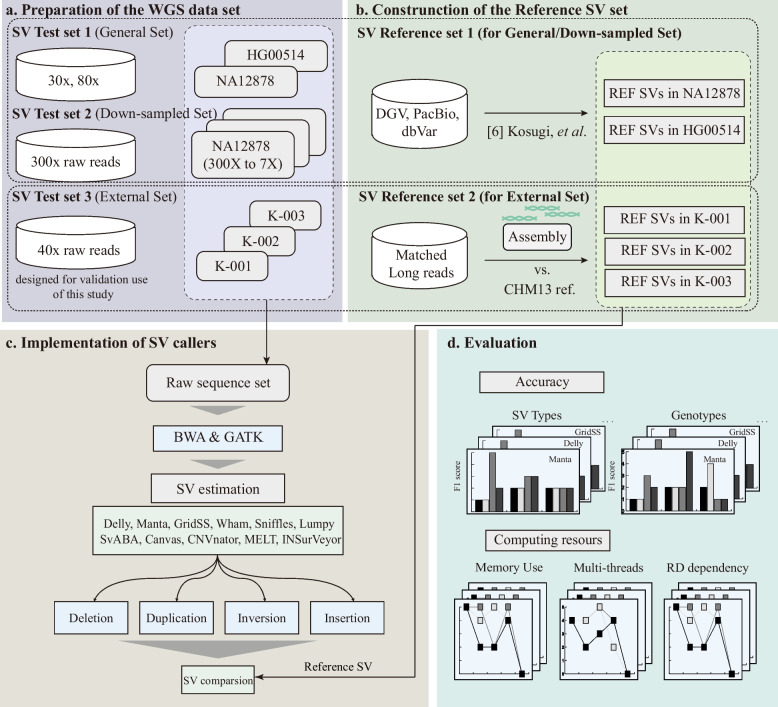
Overview of the study. **a** We constructed three whole-genome sequence (WGS) datasets: a general dataset composed of sequences for NA12878 and HG00514; a downsampled NA12878 dataset, and an external dataset with sequences derived from the collected blood samples from three participants. **b** The reference dataset for the general and downsampled dataset was composed of the structural variation (SV) data for NA128780 and HG00514 available in public datasets. The reference SV data for the external dataset were obtained through paired long-read data-based assembly. **c**, **d** For each WGS dataset, we obtained SV results according to the same pipeline and then subjected all SV types’ accuracy, computing resources, and read-depth dependency to fair comparison

This study evaluated recently published and widely used SV callers with identified variants from a massive whole-genome sequencing (WGS) dataset (Fig. 1). In this process, we selected 11 SV callers, considering the existing benchmarking results and computing power, and included tools that only consider specific types, such as CNV and mobile element insertion. To set up the reference SVs, we collected the known SVs of NA12878 and HG00514, as well as three Korean samples from long-read-based assemblies using PacBio HiFi long-read WGS data. First, we compared the overall accuracy of the SV callers and evaluated the performance of the following major SV types: deletion, duplication, inversion, and insertion. Second, we compared the computing resources, i.e., the memory usage and running time, according to the read-depth and the number of threads. In addition, we compared the estimated genotypes based on the assembly-to-assembly approach. Based on these comparisons, we discuss the methods suitable for large-cohort SV analysis and the limitations of the current SV analysis techniques. Lastly, we provide overall performance estimations for innovative SV callers.

## Results

### Reference SVs and WGS datasets

We designed a benchmarking process for SV callers to evaluate the performance of various software options. First, we prepared three WGS datasets, which were termed the general, downsampled, and external datasets (Supplementary Table S1). The general dataset consisted of one NA12878 and two HG00514 samples, the downsampled dataset consisted of one downsampled NA12878 dataset from 300 × to 7x, and the external dataset consisted of three newly generated WGS samples. Second, the reference SVs for NA12878 (HG001) and HG00514 that served as the truth sets were obtained from previously identified publications [6]. The NA12878 SVs consisted of 9241 deletions, 2611 duplications, 291 inversions, and 13,669 insertions, while the HG00514 SVs consisted of 15,193 deletions, 968 duplications, 214 inversions, and 16,543 insertions. We annotated only four types of reference SVs: deletions, duplications, inversions, and insertions. For the reference SVs of the three external sample sets, we identified new deletions and insertions using the genome assembly to assemble comparisons with the long-read approach (see Methods section).

### Comparisons of the four SV types from seven callers

**Fig. 2:**
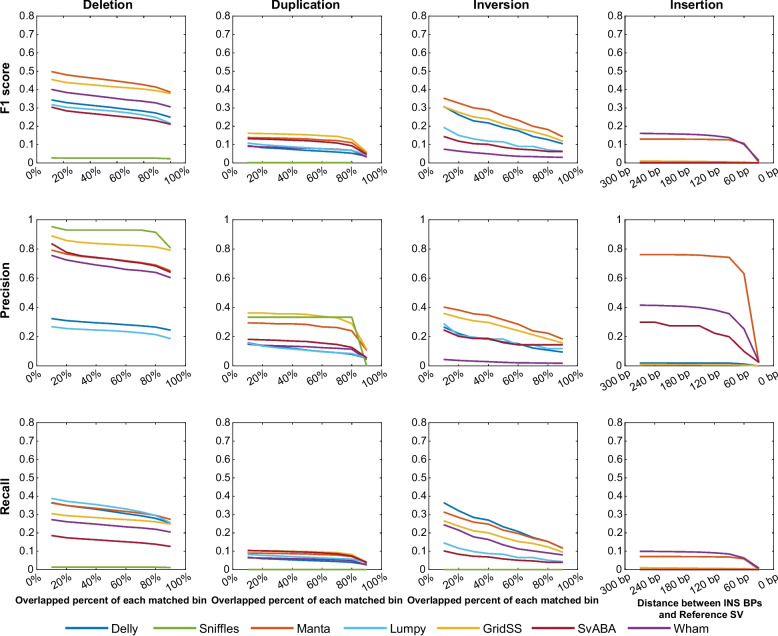
Structural variation (SV) detection performance of the seven callers. **a** For each of the four SV types, F1 scores of the seven SV tools along with the four SV types. **b**, **c** Precision and recall values of the seven SV callers according to the four SV types. Each caller was run with default parameters and a fixed length. For deletion, duplication, and inversion SVs, the SV length was restricted to at least 50 bp, and the maximum size was 1 Mbp

We selected seven widely used SV callers — Manta [15], Delly [20], GridSS [14], Lumpy [9], SvABA [21], Wham [22], and Sniffles [10] —for the efficient benchmarking study. First, we used NA12878 and HG00514 from the general dataset to investigate the accuracy of the callers. We used each SV caller with each caller’s execution pipeline’s default or recommended parameters. In general, all the SV callers successfully detected the typed SV deletions better than the other SV types (Fig. 2). For NA12878 from the general dataset, deletions that were determined using Manta were well matched in the reference dataset with the highest F1 score. The deletion precision of GridSS was > 0.9 (Fig. 2b); however, its recall rate was lower than those of the other callers (Fig. 2c).

Similarly, for Sniffles, deletions were accurately matched with a precision of approximately one. However, the recall values were significantly lower than those of the other callers; hence, the deletion detection F1 score was lower than those obtained with the other callers. For duplication and inversion detections, most callers exhibited low performance in terms of both precision and recall. Regarding insertion detection, Manta showed greater accuracy than the other callers (approximately 0.8); however, low recall values showed that Manta predicted only approximately 20% of the insertions in the reference SVs. Overall, except for Manta, the callers had insertion detection F1 scores close to zero.

Furthermore, all SV callers showed consistent performance for NA12878 and HG00514 with the general dataset (Supplementary Fig. S1) and effectively detected the deletion-type SVs; hence, these deletion-type SVs overlapped with the genomic regions on the reference SV dataset. Insertion detections by Manta showed a higher performance with an accuracy rate of 0.7 for HG00514, the same as the value obtained for NA12878. In contrast, the F1 scores for duplication detection for any of the callers was < 0.2. As we reported earlier in this section, Manta showed the best deletion detection performance of any of the callers. The precision, recall, and F1 score for Manta’s deletion calling were approximately 0.8, 0.4, and 0.5, respectively (Fig. 2b, c, Supplementary Fig. S1).

### Comparisons of SV callers according to read-depth

**Fig. 3:**
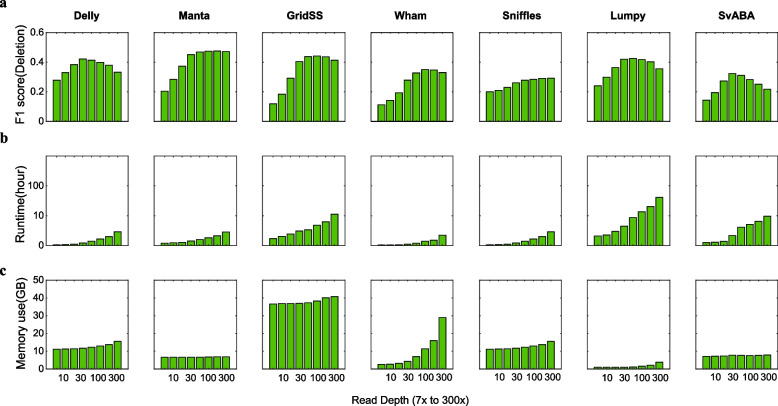
Structural variation (SV) detection performance according to the read-depth. **a** F1 scores of the seven SV callers according to read-depth. **b** and **c** The seven SV callers' run-time and maximum memory use according to read-depth. Each read-depth dataset was downsampled by random selection from the NA12878 300X dataset. The run-time figure shows the log scaled time hours

We attempted to compare the performances of various SV callers according to read-depth. For the downsampled dataset of NA12878 high-depth sequencing data, we downsampled the raw sequence data to separate the lower sequence depth using a 300 × sequence dataset. In this process, we randomly selected the sequences and constructed eight additional samples according to the read-depths, namely, 300x, 150x, 100x, 60x, 30x, 15x, 10x, and 7x (Supplementary Table S1). We then evaluated the performances of SV callers according to various depth sizes (300x, 150x, 100x, 60x, 30x, 15x, 10x, and 7x, Supplementary Fig. S2 to S9) from NA12878 to check overall SV type detection, memory use, and software run-time (Fig. 3).

When the caller conducted a depth-based investigation, the detection performance gradually increased as the depth coverage increased. However, beyond 100x, the F1 score of several SV callers tended to decrease or maintain a particular value (Fig. 3a). Furthermore, the recall value steadily increased as the depth increased; however, the precision value gradually reduced as the depth increased beyond 100x (Supplementary Fig. S10). To further explore the accuracy trade-off, we investigated by tallying the number of predicted SVs from SV callers. We observed that several SV callers made a large number of predictions as the read-depth increased. Thus, the number of both true positives and false positives increased. This increase in read-depth led to an increase in the number of true positives and a decrease in false negatives for reference SVs (see “Recall” in Supplementary Fig. S11). However, we observed that increasing the read-depth also led to an increase in false positive predictions for SVs (see “Precision” in Supplementary Fig. S11). With the exception of Wham and Sniffles, we obtained similar results across all other SV callers.

The running time and memory usage generally depend on the read-depth. For all SV caller tests, running time and memory usage were higher at a maximum depth of 300 × of the sequencing dataset than running time and memory usage for lower depth-based calling (Fig. 3b and c). Regardless of the read-depth size, we confirmed that the memory usage of Manta, GridSS, and SvABA was fixed. The memory use of the other callers tended to increase (Fig. 3c).

### Multi-thread computing test for SV callers

**Fig. 4:**
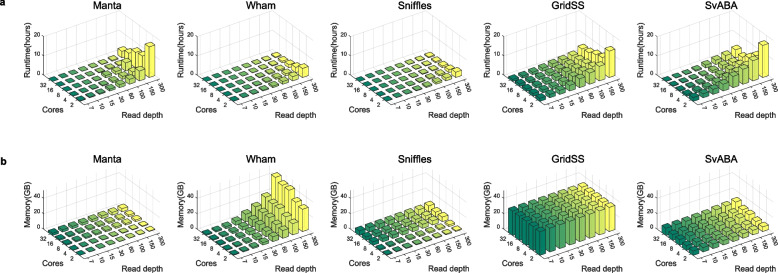
Comparison of computing performance of the SV callers according to read-depth and multi-computer processing unit threads. **a** Run-time comparison with the number of CPU threads and read-depth. **b** Maximum memory use compared with the number of CPU threads and read-depth

In the previous section, we presented the results of our tests on the performance of SV callers according to read-depth using downsampled low-depth data. For the findings detailed in this section, we additionally measured the computational resources while adjusting the number of CPU threads for time consumption and memory use confirmation using a downsampled dataset. The results for each of the five callers investigated in this portion of the study support the multi- threads option as a parameter. Therefore, we evaluated the five callers in a computing system with identical specifications. In general, when more threads are used, the run-times of most of the SV callers decreased with a multi- threads compared with fewer threads process (Fig. 4). Wham and Sniffles performed better speed than the other callers (Fig. 4a and b). When using 32 threads for processing 100x, Wham and Sniffles took approximately 30 min each.

Moreover, Manta exhibited the fastest speed improvement with increasing numbers of threads (Fig. 4a). The time consumption of GridSS and SvABA decreased as the number of threads increased, but not significantly. Overall, memory usage tended to increase as the number of threads increased, and we found that Manta and SvABA used the fewest memory resources. We confirmed that as the number of threads increased, memory usage increased most slowly in Manta and SvABA, while more memory was used in Wham and Sniffles, and the memory usage in GridSS was the highest (Fig. 4b). The run-time was positively correlated with the depth increment depending on the read-depth.

In contrast, memory usage depended more on the number of CPU threads than on read-depth. In the case of Wham, memory usage was affected by both the number of CPU threads and the read-depth. In contrast, in the case of Manta, as confirmed in the previous section, memory usage did not increase regardless of whether the depth increased. Unlike that of Manta, the memory usage of Sniffle and SvABA increased slightly.

### Comparisons with accurate assembly-based SVs and genotypes

To overcome the limitation of an accurate reference SV dataset, we designed a long-read-based SV reference dataset from the three individual samples to further validate the SV callers. We used paired short- and long-sequence datasets as the external dataset in these comparisons. In this process, we constructed a HiFi long-read dataset for the three new samples, including both the short- and long-read WGS data. After completing the three assemblies using the long-read data, we identified the reference SVs using the assembly-to-assembly approach. Finally, we obtained approximately 30 K deletions and 30 K insertions as reference SVs for each assembly.

**Fig. 5:**
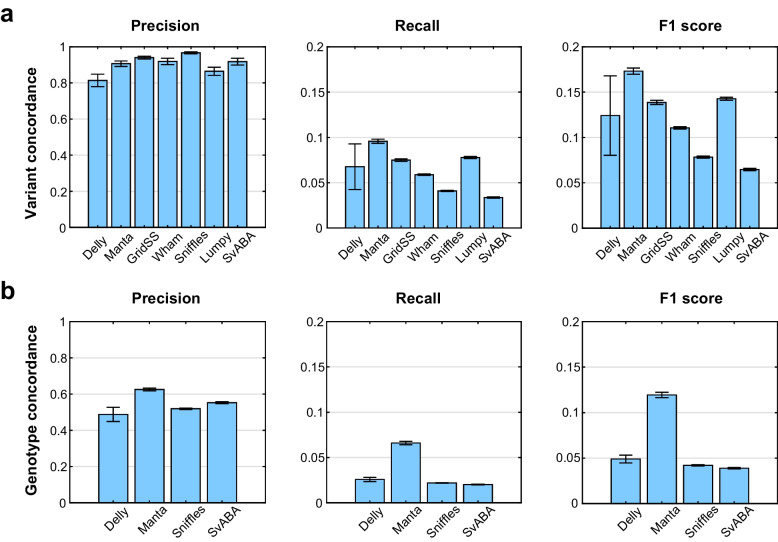
Structural variation detection performance for the seven structural variation (SV) callers with long-read-based assembly data. **a** The performances of detected deletions in three additional samples with long-read reference SVs. **b** The performances of genotype concordances with long-read reference SVs. We selected SVs with at least 50% overlap length

Next, for comparison with short-read datasets, we similarly applied the seven SV callers with each matched short-read dataset based on the long-read SV reference dataset. SvABA, Sniffles, and GridSS showed superior precision in deletion detection, whereas Manta showed higher recall and F1 scores than other callers (Fig. 5a). Most SV callers showed high precision in the detection of deletions; however, insertion detection was poor, similar to what we observed for NA12878 and HG00514. Only Manta showed higher accuracy in insertion detection. Notably, Sniffles and SvABA showed very high precision (> 0.9) in deletion detection, and Manta showed approximately 0.9 precision in the detection of both deletions and insertions. We verified approximately 90% of the detected SVs from those callers in the long-read-based reference SVs (Fig. 5). In addition, we examined the genotyping prediction using the estimated results of each caller which was able to predict variant genotypes; hence, we ascertained the genotype concordances by comparing SVs obtained from the callers with the results of assembly-based reference SVs from our in-house process. The genotype distribution of SVs was similar to that of the assembly-based reference results. In the case of deletions, both Manta and Delly showed the highest genotype concordances (Fig. 5b). Nevertheless, Sniffles and SvABA accurately reported matched genotypes in only a small number, similar to previous variant concordances. In the case of insertions, the capability of Manta SV was the best, and the insertion performance of the others was relatively low, complicating comparisons.

### Additional large-scale range SVs and mobile element insertion

**Fig. 6:**
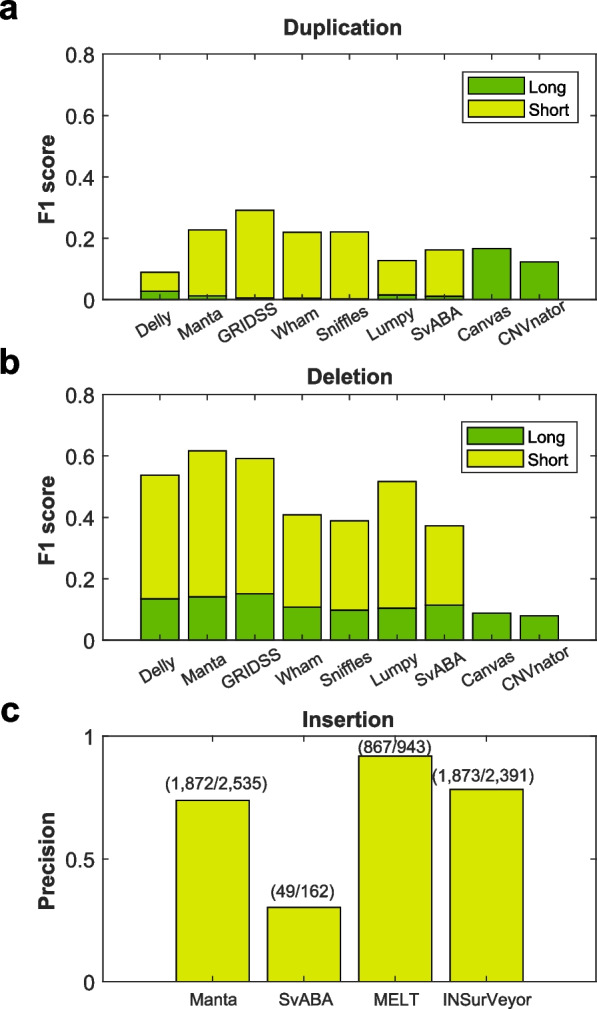
Performance of long duplication/deletion and insertion specific detection by Canvas, CNVnator, MELT and INSurVeyor. Selected structural variations (SVs) with at least 50% overlap length from NA12878 (60 × dataset). **a** The performance of SV callers for duplication detection according to SV length. **b** The performance of SV and copy number variation (CNV) tools for deletion according to SV length. Short size: > 50 bp and < 1 Kbp. Long size: > 10 Kbp. **c** The performance of insertion detection with MELT and INSurveoyr. The numbers in the parentheses represent the number of reference SVs and the number of SVs estimated by each tool

Finally, we evaluated the copy number detection tools for detecting long-range SVs. Because a read-depth-based calling process is more accurate for searching long duplications, we added read-based tools for long-range SV detection. In this process, we evaluated the CNV detectors called Canvas [23] and CNVnator [24]. Concerning detecting duplications > 1 kb, Canvas and CNVnator clearly showed higher precision and recall than the other SV detection tools (Fig. 6a). Generally, for most SV types, long-range SVs are less likely to be identified than short-range SVs. However, read-depth-based tools, such as Canvas or CNVnator, showed improved detection regarding long duplications. In addition, the precision and recall for deletion detection of CNV tools were similar to those of the SV callers we evaluated (Fig. 6b). As described in the previous sections, most SV callers showed relatively lower calling performance in insertion than in deletion. Hence, we investigated only the detection of the insertions of mobile elements using the MELT [25] and INSurVeyor [26] callers and compared its performance against those of the other SV callers. The MELT caller showed the highest insertion detection precision, at approximately 0.9, which was better than the value obtained with Manta (Fig. 6c). MELT only focused on the insertions of mobile elements; however, often, insertions in genomes are those of the mobile elements. Hence, MELT results can be used more effectively to determine the insertion SV loci. Additionally, INSurVeyor showed the also slightly better precision than Manta for insertion detection.

## Discussion

Since the advent of NGS technology and the availability of several computational methods, the search for SVs in the genome has increased dramatically. However, various limitations are presented, such as read length and sufficient depth. We used short-read-based data to compare nine tools (Delly, Manta, GridSS, Wham, Sniffles, Lumpy, SvABA, CANVAS, and CNVnator) according to the coverage depth for NA12878 and HG00514. We then evaluated the detection performance of the four types of SVs. Most detections were from read pairs, split-reads, read-depths, and local assembly [27]. Manta uses graph-based algorithms to detect SVs from next-generation sequencing data. It's designed for rapid and accurate detection of SVs, particularly deletions and translocations, by analyzing paired-end reads and split-read evidence with assembly-approach. Delly employs a combination of paired-end and split-read methods. It utilizes paired-end mapping and read-depth signal analysis for SV discovery. GRIDSS uses a break-end assembly approach to detect structural rearrangements. The method involves identifying discordant read pairs and split-reads, then assembling them into longer sequences to pinpoint structural variations. Wham uses a graph-based approach to analyze next-generation sequencing data for SV detection. Sniffles is designed specifically for long-read sequencing data. It uses a read-alignment-based approach to detect and classify SVs. Lumpy uses probabilistic models to integrate multiple SV detection signals, including read-pair, split-read, and read-depth. SvABA is a structural variant caller that uses local assembly and multiple alignment strategies to detect SVs and indels. Canvas is designed for copy number variation (CNV) detection. It uses a read-depth approach, analyzing coverage data from whole-genome or targeted sequencing. Canvas is efficient in detecting large-scale CNVs. CNVnator also focuses on CNV detection using a read-depth method, but it employs a unique approach by dividing the genome into fixed-size bins and analyzing read-depth within these bins to identify CNVs. Mobile Element Locator Tool (MELT) specializes in detecting mobile element insertions in genomic data. It uses a combination of split-read and paired-end data to identify insertions of transposable elements. INSurVeyor focuses on insertion detection. It analyzes split-read data and uses a pattern recognition approach to identify and characterize insertions, including those of novel sequences.

Manta, GridSS, and SvABA are based on read-pairs, split-reads, and local-assemblies, whereas Delly and Lumpy are based on read-pairs and split-reads [28]. The local assembly method significantly improves novel insertion and small, highly rearranged SV detection. These SV callers have limitations in accurately detecting SVs > 1 Mbp, and the split-read-based method performs well in detecting smaller SVs (50–100 bp). Hence, ultra-deep sequencing data with read-depths of over 100 × could give low precision and sensitivity for deletion, as shown in Fig. 3. In addition, for evaluation based on computing resources, we checked the memory usage and operation speed depending on the number of CPU threads for five types of SV callers (Manta, Wham, Sniffles, GridSS, and SvABA), which are software types capable of parallel operation. Manta, suitable for numerous large-scale cohorts, showed notable performance in terms of computational speed and memory usage.

Our analysis of the computing performance of various SV callers revealed significant insights into their efficiency and resource utilization. Overall, it appears that these tools are increasingly being optimized to reduce processing time, a crucial factor in large-scale genomic analyses. Wham's Performance with Increased Cores: We observed that Wham benefits from an increase in core count, resulting in enhanced processing speed. However, this comes at the cost of increased memory consumption. This trade-off is an important consideration for users with limited memory resources or when processing particularly large datasets. For other tools in our study, we noticed a trend where memory usage increased with higher read-depth rather than with the number of cores. This suggests that memory allocation is more closely tied to the complexity and size of the data being processed than to the sheer processing power. A notable advantage was seen with GridSS, which allows users to adjust memory usage through parameters. This flexibility can be particularly advantageous in environments where memory resources are a limiting factor, enabling more effective management of computational resources.

Evaluating most of the SV callers in quantitative terms of performance was difficult, except regarding deletion detection performance. Moreover, Manta SV exhibited relatively high performance in terms of the detection of insertions. In the case of long duplications, the accuracy of CNV tools, such as CNVnator and Canvas, was confirmed based on read-depth approaches. Long-size duplication detection was a strength of this copy number detection approach. Moreover, we performed an interesting comparison for a more accurate validation at the end of this study. In this study, we constructed three additional genomes with long-read and short-read sequences produced from the same samples, called the external dataset. The performance results of the short-read-based SV callers were verified by the results found on long-read-based SV. The performance of deletion detection in most of the SV callers with our matched short-read and long-read datasets was consistent with public data; however, in the case of precision (i.e., number of true SVs/obtained SVs), we confirmed improved performance. Notably, we performed a genotyping test from the phasing operation of the long-read-based genome assembly. Because most callers provide estimated values of heterozygous and homozygous genotypes, we thought these results should also be considered. We, therefore, assessed genotypes on assemblies generated from a sample compared with those from the SV callers. In the case of deletions, we confirmed > 50% genotype concordance using most callers, and only Manta showed > 60% precision concordance with long-read reference SVs. Performance for insertion detection in the estimated genotype was higher in Manta than in the others.

When comparing various depth coverages, we observed a decrease in the F1 score for some of the SV callers when the depth of coverage was increased. Upon further investigation, we found that with higher coverage, true positives and false positives may both increase, resulting in a trade-off between recall and precision. Therefore, the choice of coverage depends on the specific purpose of the study. In terms of computing resources, Manta, GridSS, and SvABA showed fixed memory usage that depends only on the number of threads, making them consistent when using datasets with varying depths of coverage. We confirmed that Manta and SvABA demonstrated high memory efficiency, even in higher-depth datasets, such as 300x.

Furthermore, we validated SV callers in three independently constructed WGS datasets, and the results were consistent with those on NA12878 and HG00514. However, in our additional comparison of insertion SVs, MELT had the highest precision for insertion detection, while Manta detected almost twice as many insertions despite having a slightly lower accuracy score. Therefore, the choice of the tool depends on the specific needs and goals of the study. In some cases, detecting more insertions may be more important than slightly lower accuracy, while in other cases, the opposite may be true.

We also extended our analysis to include synthetic datasets with 30 × and 60 × coverage, to which we introduced arbitrary SVs. This approach enabled us to perform additional testing that goes beyond the limitations of our constructed reference SVs. Notably, in these simulations, we observed a higher accuracy in deletion detection within the simulated data compared to the real data (Table S2). This observation is particularly enlightening as it underscores the robustness and effectiveness of most SV calling algorithms in controlled, simulated environments. However, it also brings to light the significant challenges these algorithms face when applied to real genomic data. The complexity and intricacy of actual genomes often present more nuanced and challenging scenarios for SV detection, which are not fully captured in simulated conditions.

In this study, although we attempted to comprehensively compare SV callers based on various aspects, there were certain limitations. First, we used only two public samples to ensure relevant reference SVs that were gold-standard. However, for more relevant and consistent results, larger-scale reference samples such as those from the 1000 Genome Project could be used. Second, there are various and complicated types of SVs, including translocations, which are important in genetic diseases such as cancer or rare disease. However, the task of identifying a golden-standard reference set of SVs was arduous and continues to be challenging. The relevant reference SVs previously revealed only consist of deletion, insertion, duplication, and inversion, so we focused on these four types of SVs which can be compared with each SV callers. Additionally, highly accurate deletion insertion variants were suggested to us through long-read assembly-based experiments, which we used for validation. Nonetheless, a lack of various and complicated types of SVs remains, including duplication, inversion, and translocations. We note that the development of accurate and reliable golden standard SVs will lead to more consistent and relevant SV caller comparison results. Here, we analyzed the detection performance of the SV callers for deletions and insertions with short-read-based WGS data by constructing a matched long-read WGS dataset. In contrast to previous benchmarking studies that employed varying callers and diverse simulation datasets [6, 16], our primary focus was on computing resources according to depth and multi-thread computing in massive WGS datasets. Additionally, we compared major SV callers by using long-read sequencing to identify reference SVs and evaluated the performance of each caller by matching with the short-read dataset. This benchmark study may prove helpful as a reference for applying SV callers and computing resources in genomic studies of large-scale populations.

## Conclusions

Our findings consistently indicate Manta as a highly effective and versatile SV caller across various types of structural variations. Its balanced approach to accuracy, speed, and memory efficiency makes it a robust choice for a wide range of SV detection tasks. However, our study also underscores the importance of selecting specialized tools for certain SV types. For instance, in cases of long duplications, depth-based CNV callers demonstrate superior performance. These tools are better equipped to handle the nuances associated with large-scale copy number variations. Similarly, for insertion events, tools like MELT or INSurVeyor offer more refined detection capabilities, making them a preferable choice for such specific SV analysis. Recognizing the strengths and limitations of individual SV callers, we are exploring the potential of ensemble methods. These methods would integrate the diverse capabilities of various tools, aiming to improve overall accuracy and comprehensiveness in SV detection. Such an approach could be particularly beneficial in complex genomic landscapes where a single tool may not capture all aspects of structural variation. Our research also highlights the inherent limitations in detecting certain types of SVs, particularly with short-read sequencing data. While tools like Manta perform well in identifying deletions, the detection of more complex SVs remains challenging. This limitation is primarily due to the constraints in read length and depth inherent to short-read sequencing technologies. We emphasize the growing importance of long-read sequencing in overcoming these challenges. Long-read technologies offer significant advantages in resolving complex SVs, and continued advancements in this field are likely to catalyze the development of more sophisticated SV calling algorithms. Our future research will focus on leveraging long-read data to enhance the detection and interpretation of complex structural variations.

Large-scale genomic projects are underway worldwide to develop precision medicine and early diagnostic technologies. These projects produce in-depth whole-genome data to identify the causes and ensure early diagnosis of rare or ethnicity-specific diseases. With the development of long-read sequencing technology, interest in information on SVs that have a more significant effect on pathogenesis than single nucleotide polymorphisms or short indel variants have is increasing rapidly. To analyze SVs from massive WGS data, researchers should consider the computing requirements of SV callers, such as time consumption and memory capacity. Accuracy is an essential feature for determining SVs.

In conclusion, considering the execution time, memory usage, and accuracy, Manta-based SV detection depends on large-scale WGS data, including genotype estimations. However, most SV callers showed a relatively low performance for other types of SVs, including duplication and inversion. In particular, the read-depth-based copy number detection tool showed good performance in the case of long duplications. In the case of insertions, a search tool specialized for mobile elements appeared more effective. Notwithstanding, identifying various types of SVs is still challenging, and we believe this study will assist in defining SVs with sizable WGS data from a large-scale population.

## Methods

### Public genome dataset for benchmarking

We used three WGS datasets: the general, downsampled, and external datasets (See Supplementary Table S1). For the general dataset, we used one NA12878 and two HG00514 samples whose raw datasets were downloaded from the National Center for Biotechnology Information Sequence Read Archive (SRA, https://www.ncbi.nlm.nih.gov/sra). For the downsampled dataset, we used the high-depth sequenced NA12878 dataset obtained from The International Genome Sample Resource (https://www.internationalgenome.org/data-portal/sample); this dataset was downsampled to benchmark the SV caller test according to read-depth using seqtk (version 1.3, https://github.com/lh3/seqtk). Additionally, to create simulated datasets for evaluating the performance of structural variant callers, we utilized VarSim (version 0.8.5, –sv_num_ins 20,000 –sv_num_del 20,000 –sv_num_dup 4000 –sv_num_inv 4000 –sv_percent_novel 0.01), available at https://github.com/bioinform/varsim. With this raw sequence and SV generation tool, we generated synthetic raw datasets at 30 × and 60 × coverage with 100 bp paired-end reads.

### Sample preparation and DNA isolation for the external dataset

We generated three new sequenced genomes from a part of the National Genome Project that were paired with long- and short-read datasets. Blood was collected from three healthy Korean individuals (K-001: female, aged 50 years old; K-002 and K-003: males, aged 29 and 36 years old, respectively). The samples were obtained from Chungnam National University Hospital (Daejeon, Republic of Korea). Genomic DNA was then isolated from 5 mL of blood using a DNeasy Blood & Tissue Kit, according to the manufacturer’s instructions (Qiagen, Carlsbad, CA, USA). The quality and quantity of extracted genomic DNA were analyzed using an ND-1000 spectrophotometer (Thermo Fisher Scientific, Waltham, MA, USA).

### WGS library preparation and sequencing for the external dataset

We used the MGIEasy FS DNA Prep kit (MGI) for short-read library construction according to the manufacturer's instructions. We then used a DNBSEQT7 sequencer (MGI) which was run on the 150 bp paired-end sequencing mode. For long-read sequencing, we used the Sequel II HiFi system (Pacific Biosciences, CA, USA). HiFi sequencing libraries were prepared and immediately treated with the Enzyme Clean Up Kit (Pacific Biosciences, CA, USA). After pooling the desired size fractions, the final libraries were cleaned and concentrated using AMPure PB beads (Pacific Biosciences, CA, USA). Finally, all library concentrations were measured using a Qubit™ 1X dsDNA HS Assay Kit (Thermo Fisher, MA, USA) and then sequenced using a Sequel II HiFi system.

### Raw WGS data preprocessing

We trimmed public and in-house short-read-based WGS datasets using Cutadapt (version 3.5 with Python 3.8.4) [29] with read lengths of at least 70 bp. BWA (version 0.7.15) software [30] was used with default parameters for read mapping, and the GATK (version 4.2.4.1) [31, 32] pipeline was used for all short-read data. We filtered out duplicates and applied base quality score recalibration with GATK. For the general and downsampled sets, we used the human genome GRCh38 (resources_broad_hg38_v0_Homo_sapiens_assembly38.fasta from https://console.cloud.google.com/storage/browser/genomics-public-data/resources/broad/hg38/v0) and for the assembly-based external set, we used T2T-CHM13v2.0 [33] as the reference genome.

### Long-read-based de novo genome assembly and reference SVs

For the NA12878 and HG00514 reference SVs, we downloaded four SV types previously identified in the study of Kosugi et al. [6]. Additionally, we identified long-read-based SVs using three HiFi long-read datasets. In this process, HiFi reads from the three Korean blood samples were assembled de novo and phased using hifiasm [34] (version 0.16.0; default settings). We prepared the reference genome by merging the CHM13 genome [33] (draft v2.0; https://s3-us-west-2.amazonaws.com/human-pangenomics/T2T/CHM13/assemblies/chm13.draft_v2.0.fasta.gz) and computed high-frequency k-mers in the reference genome using meryl in Winnowmap [35] (version 2.03; meryl count k = 19 and meryl print greater-than-distinct = 0.9998). We then aligned the six phased Korean genome assemblies to the reference genome using Winnowmap (version 2.03; winnowmap -W k-mer.file.txt -ax asm20 –cs -r2k) and sorted and indexed the output BAM files using SAMtools (version 1.13; samtools sort -m4G and samtools index). Finally, we called the structural variants of each assembly using SVIM-asm (version 1.0.2; SVIM-asm haploid).

### SV caller implementation with WGS datasets

We obtained all SV callers from public open-source websites. These tools and websites are listed up in Supplementary Table S3. To conduct SV detection, seven callers were used for the multiple type detection test: Delly [20] (version 1.1.8), Manta [15] (version 1.6.0), GridSS [14] (version 2.13.2), Wham [22] (version 1.8), Sniffles [10] (version 2.2), Lumpy [9] (version 0.3.1), and SvABA [21] (version 1.2.0), as well as two CNV callers, CNVnator [24] (version 0.4.1) and Canvas [23] (version 1.4.0) for CNV and the mobile element detection caller, MELT 32] (version 2.2.2) and INSurVeyor [26] (version 1.1.2) or insertion specific. We used these callers with default parameters and filtered the final SV results accordingly. The results of the SV callers were manually converted for proper SV annotations (i.e., DEL, DUP, INS, and INV). For DEL, DUP, and INV, SVs were selected with a length of at least 50 bp and a maximum length of 1 Mbp.

### SV evaluation rules using the reference SVs

To assess the metrics of precision and recall, we adjusted the partially matching rules. To determine precision, we counted the number of true positives from the predicted SVs that partially or completely matched the reference SVs reciprocally. The matched rate or percentage was calculated based on the size of the overlapping base pairs (bp). For example, if there is an SV with a length of L' bp predicted by an SV caller and there is an overlapped region of length L bp compared to a reference SV with a length of L'', we identified the matched ratio as the minimum of L/L' and L/L'' (i.e., reciprocally matched ratio). If no matched SVs were found compared to the reference SV, we calculated the matched ratio as zero. We calculated the matched ratio for all predicted SVs and counted true positives based on the matched ratio or percentage at each threshold. SVs with matched ratios less than the threshold were assigned as false positives. To assess recall, we similarly counted true positive SVs that had a matched ratio or percentage from the reference SVs. If the length of the reference SV is L'' bp and there is a predicted SV with a length of L', we calculated L bp as the overlapped region. We counted true positive in the reference SVs if the minimum between L/L' and L/L'' is greater than the threshold. If the minimum of L/L' and L/L'' is less than the threshold, we counted this reference SV as a false negative. To ensure a fair comparison, we separately calculated precision and recall according to the predicted and reference SVs and compared the F1 score, which is the harmonic mean of precision and recall between all tested SV callers. For the comparison analysis with computing resources, long range SV, and assembly-based SV analysis, we used matching percent as 50%. The details are explained in Supplementary Fig. S12.

### SV caller computing cost and time consumption

We used partially or entirely matched SVs to compare how accurately and how efficiently SVs were detected for all types according to read-depth, memory-use, and time consumption. We adjusted the read-depth size by downsampling the 300 × NA12878 genome sequences and evaluated each caller’s performance. Simultaneously, we changed the number of CPU multi-thread if a caller offered multi-thread parameter and estimated each SV caller’s memory use and run-time. In this process, our computing instrument was an AMD Opteron (TM) Processor 2.3 Ghz 32 Core with 320 GB RAM.

### Evaluation of the SVs with estimated genotypes from callers

We used three phased long-read data-based assemblies to evaluate the genotyping performance of each caller. Each pair of assemblies was built using three samples, resulting in six assemblies. The SV genotypes (GTs) were determined based on each phased assembly and were used as reference SVs. To evaluate each genotype, i.e., 1/0 or 1/1, we verified that the SV GT for each caller was found in only one or both reference assemblies.

### Supplementary Information

**Supplementary Material 1.**

## Data Availability

The original contributions presented in the study are included in the article and Supplementary Material. The external sequencing datasets produced in this study are deposited in the Korean Nucleotide Archive (KoNA) (https://www.kobic.re.kr/kona/; BioProject ID: KAP220172) and Korea BioData Station (K-BDS) [36]. However, the data underlying this article cannot be shared publicly due to Korea Bioethics and Safety Act. The data will be shared on reasonable request to the corresponding author or the Korean Nucleotide Archive (KoNA) team (data@kobic.kr).

## Abbreviations

CNV: Copy number variation
CPU: Computer processing unit
DEL: Deletion
DUP: Duplication
GT: Genotype
INV: Inversion
INS: Insertion
NGS: Next-generation sequencing
SRA: Sequence read archive
SV: Structural variation
T2T: Telomere-to-telomere
WGS: Whole-genome sequencing
