# Supplementary material for: Comparison of structural variant callers for massive whole-genome sequence data
Source: BMC Genomics. 2024 Mar 28;25:318. doi: 10.1186/s12864-024-10239-9 (PMC10976732; doi:10.1186/s12864-024-10239-9)
Supplement: Supplementary file 1 — Supplementary Material 1. [file 12864_2024_10239_MOESM1_ESM.docx]

# **Supplementary Materials**

# **Comparison of structural variant callers for massive whole-genome sequence data**

Soobok Joe^1^, Jong-Lyul Park^2^, Jun Kim^3^, Sangok Kim^1^, Ji-Hwan Park^1,4^, Min-Kyung Yeo^5^, Jin Ok Yang^1,6,*^ and Seon-Young Kim^1,4,*^

^1^Korea Bioinformation Center (KOBIC), Korea Research Institute of Bioscience and Biotechnology (KRIBB), Daejeon 34141, Republic of Korea

^2^Aging Convergence Research Center, Korea Research Institute of Bioscience and Biotechnology (KRIBB), Daejeon 34141, Republic of Korea

^3^Department of Convergent Bioscience and Informatics, College of Bioscience and Biotechnology, Chungnam National University, Daejeon 34134, Republic of Korea

^4^Department of Bioscience, University of Science and Technology (UST), Daejeon 34113, Republic of Korea

^5^Department of Pathology, Chungnam National University School of Medicine, Daejeon 35015, Republic of Korea

^6^Department of Bio and Brain Engineering, Korea Advanced Institute of Science and Technology (KAIST), Daejeon 34141, Republic of Korea

***Co-corresponding authors**: Dr. Seon-Young Kim (e-mail: kimsy@kribb.re.kr; Tel: +82-42-879-8500); Jin Ok Yang (e-mail: joy@kribb.re.kr, telephone: +82-42-879-8550)


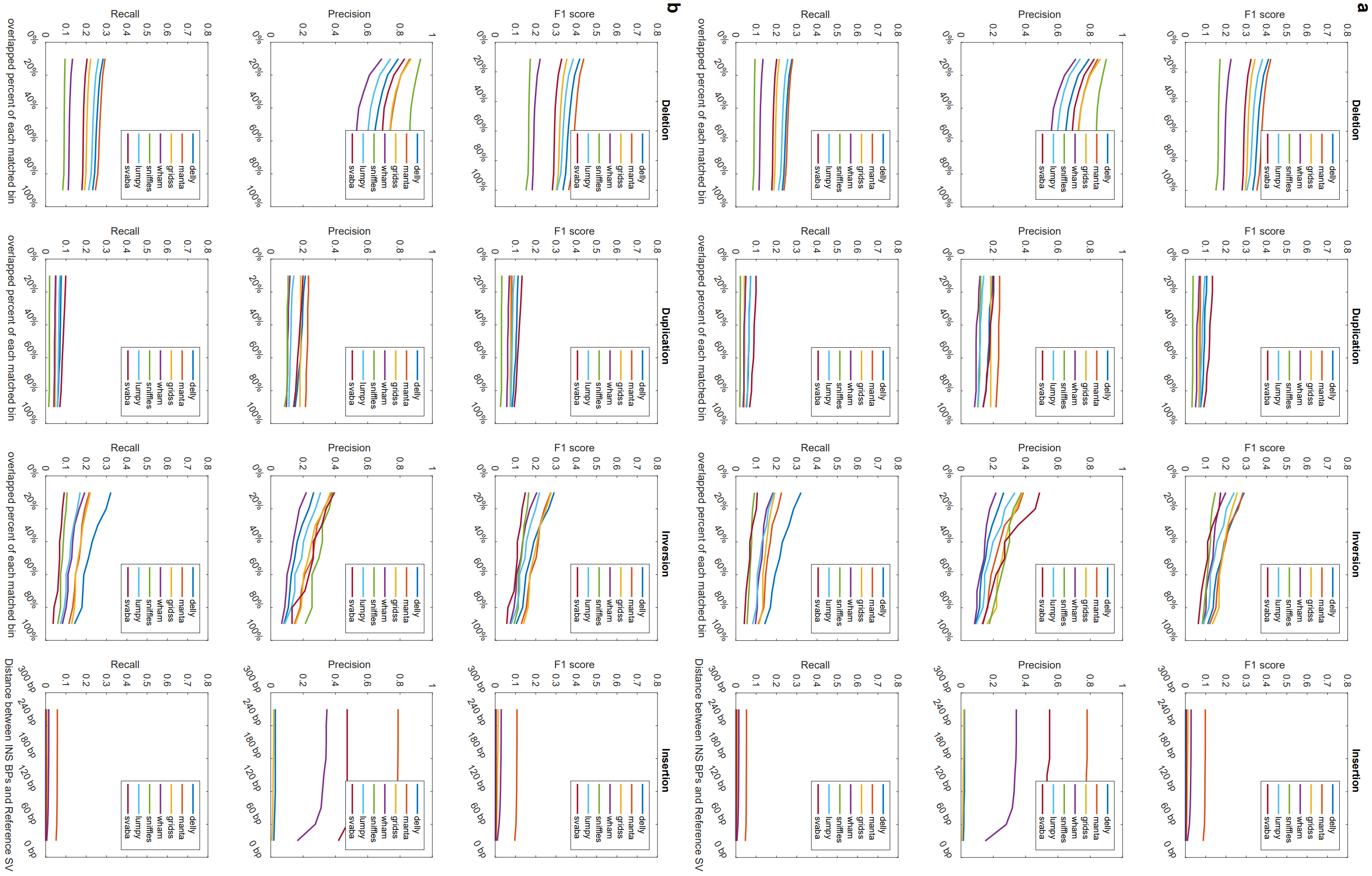


**S1 Fig. Structural variation (SV) detection performance of seven callers in HG00514.** a. General set of HG00514 with ERR894729. b. General set of HG00514 with ERR903030.

**
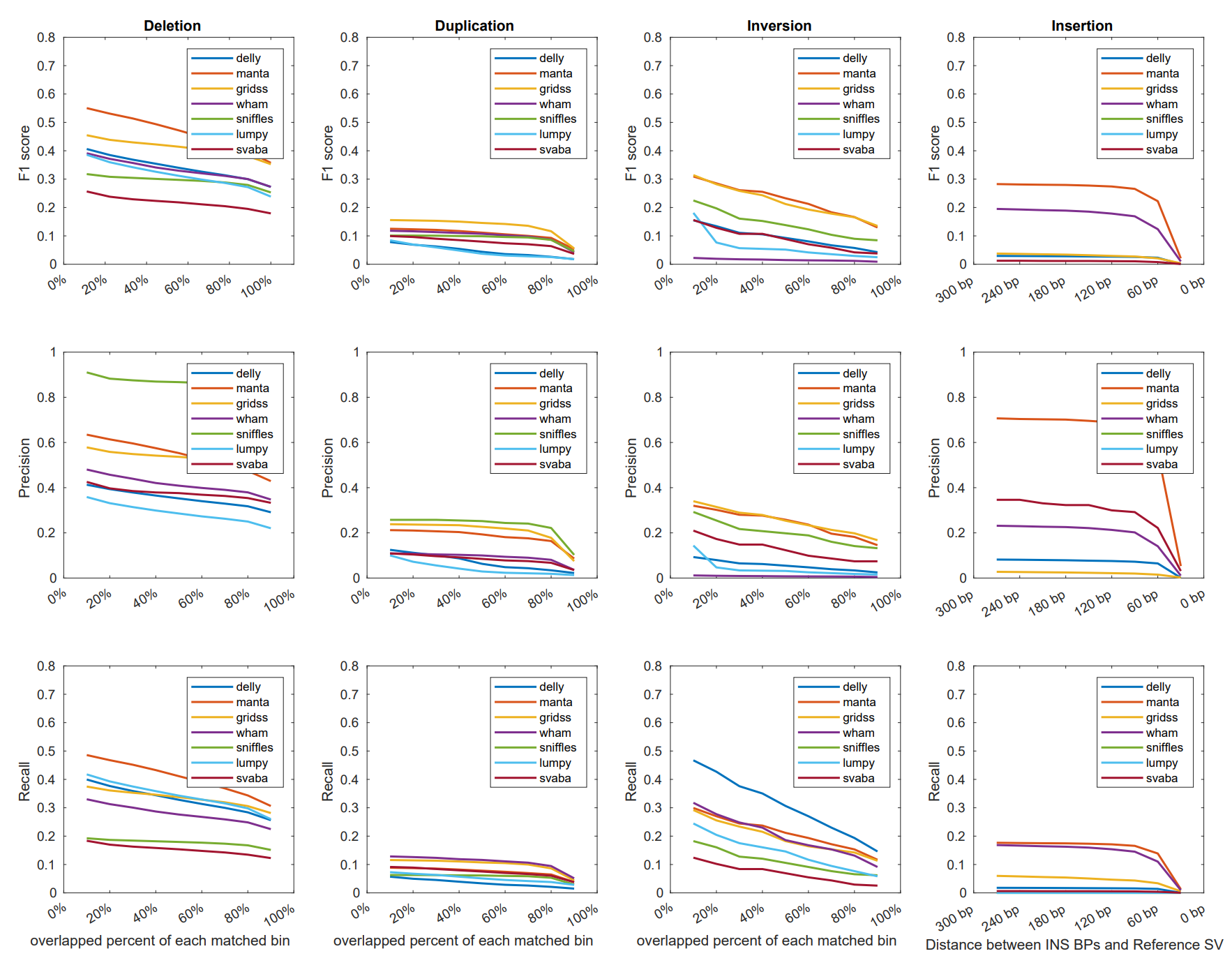
**

**S2 Fig. Structural variation (SV) detection performance of seven callers in 300x NA12878.**

**
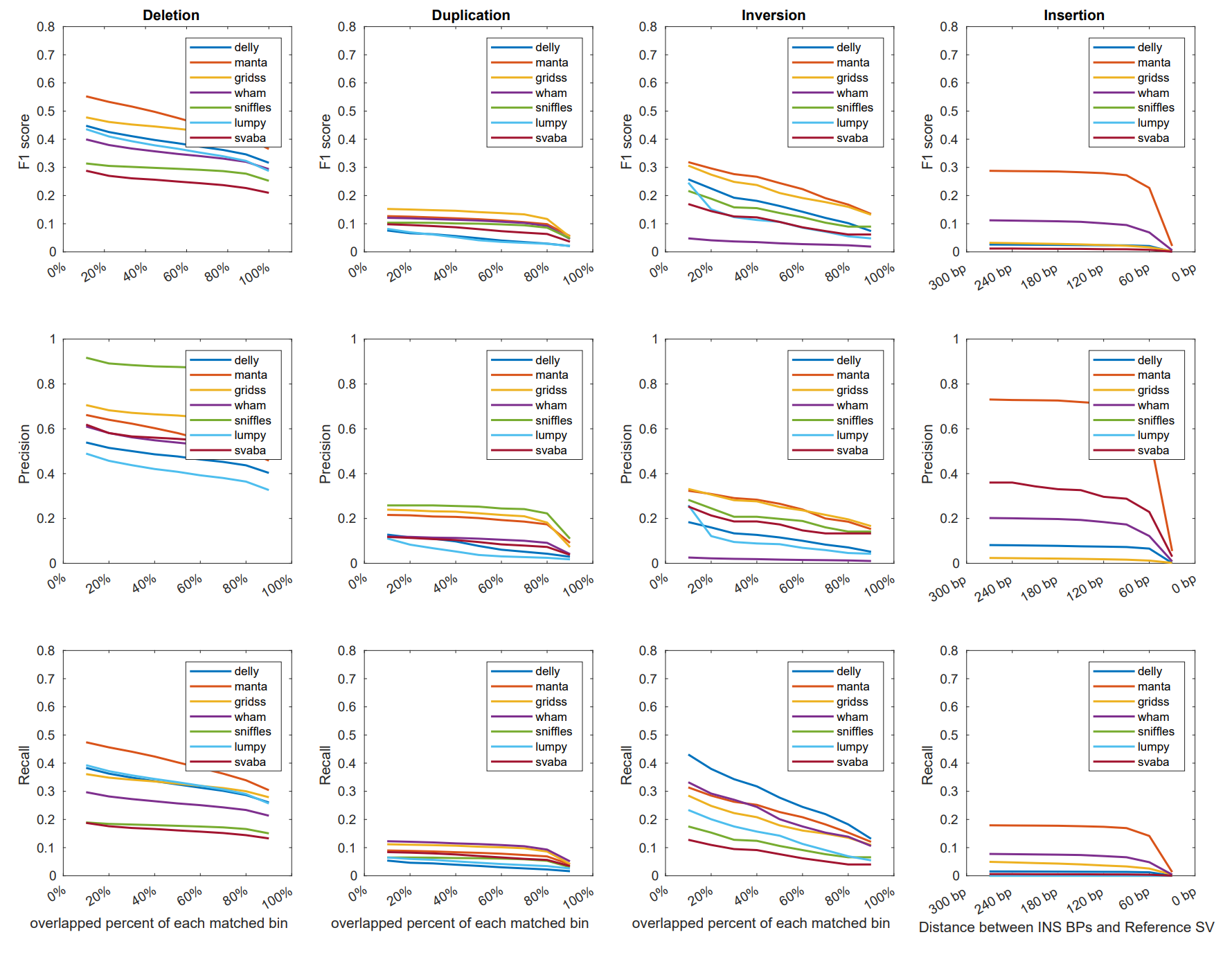
**

**S3 Fig. Structural variation (SV) detection performance of seven callers in 150x NA12878.**

**
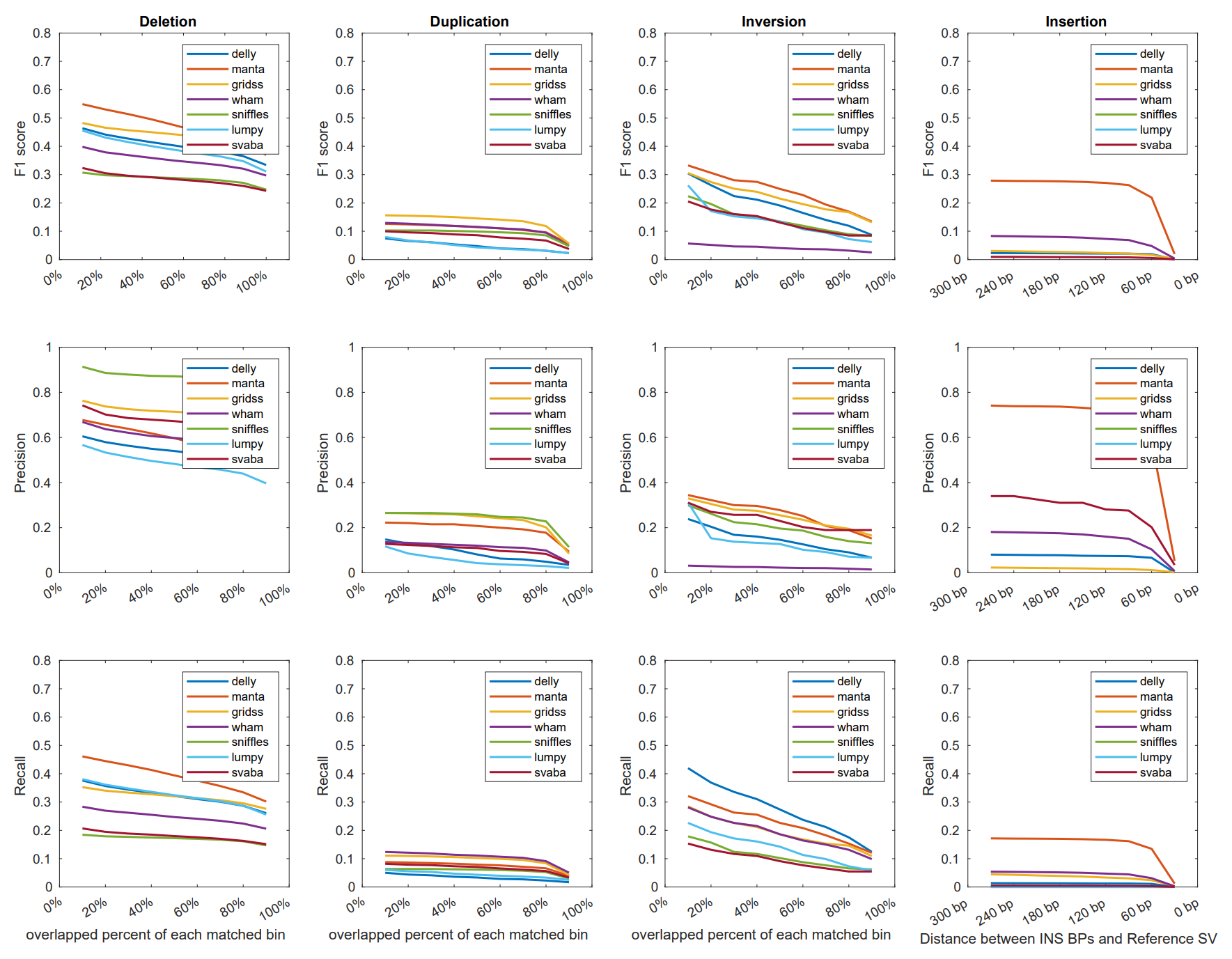
**

**S4 Fig.** **Structural variation (SV) detection performance of seven callers in 100x NA12878.**

**
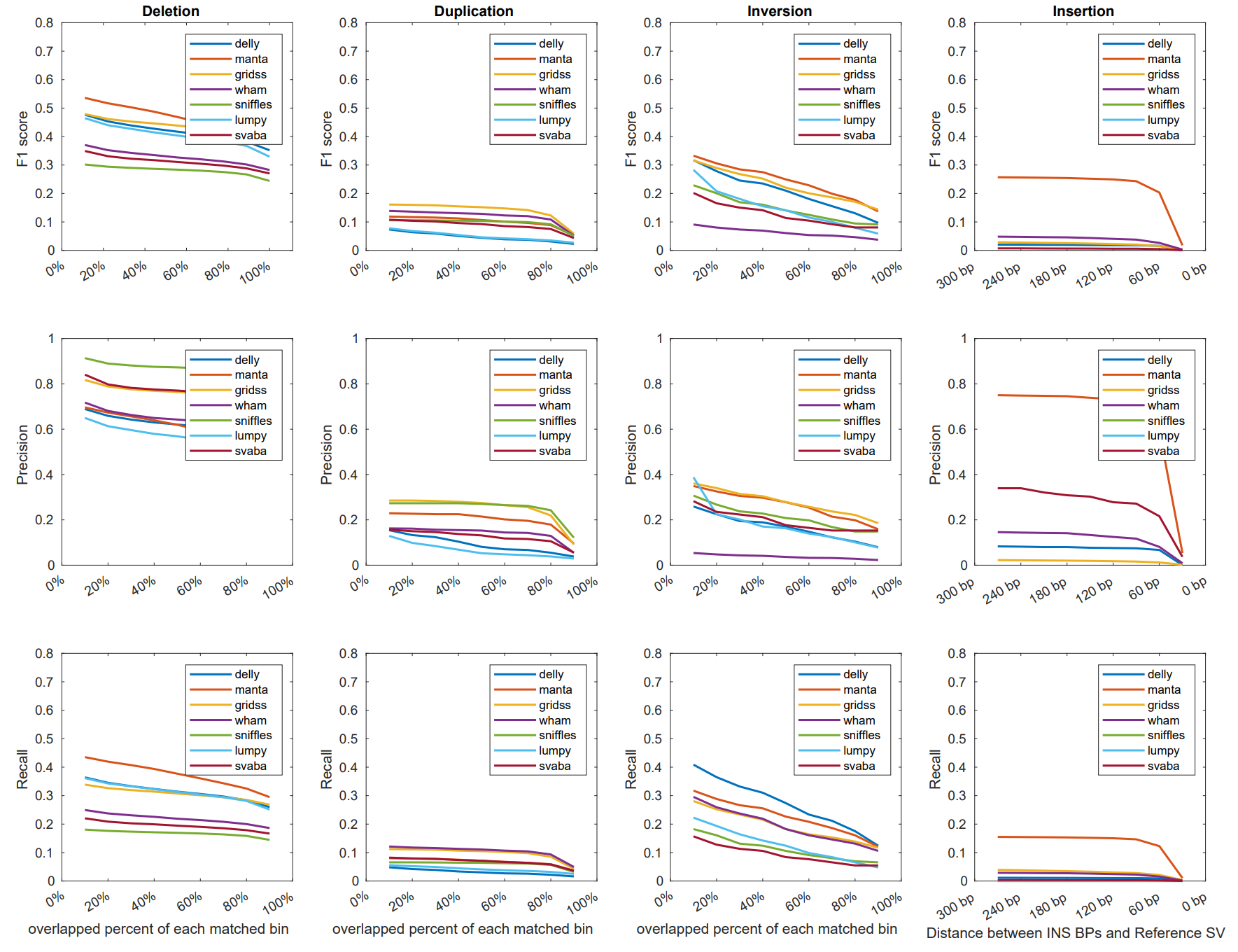
**

**S5 Fig.** **Structural variation (SV) detection performance of seven callers in 60x NA12878.**

**
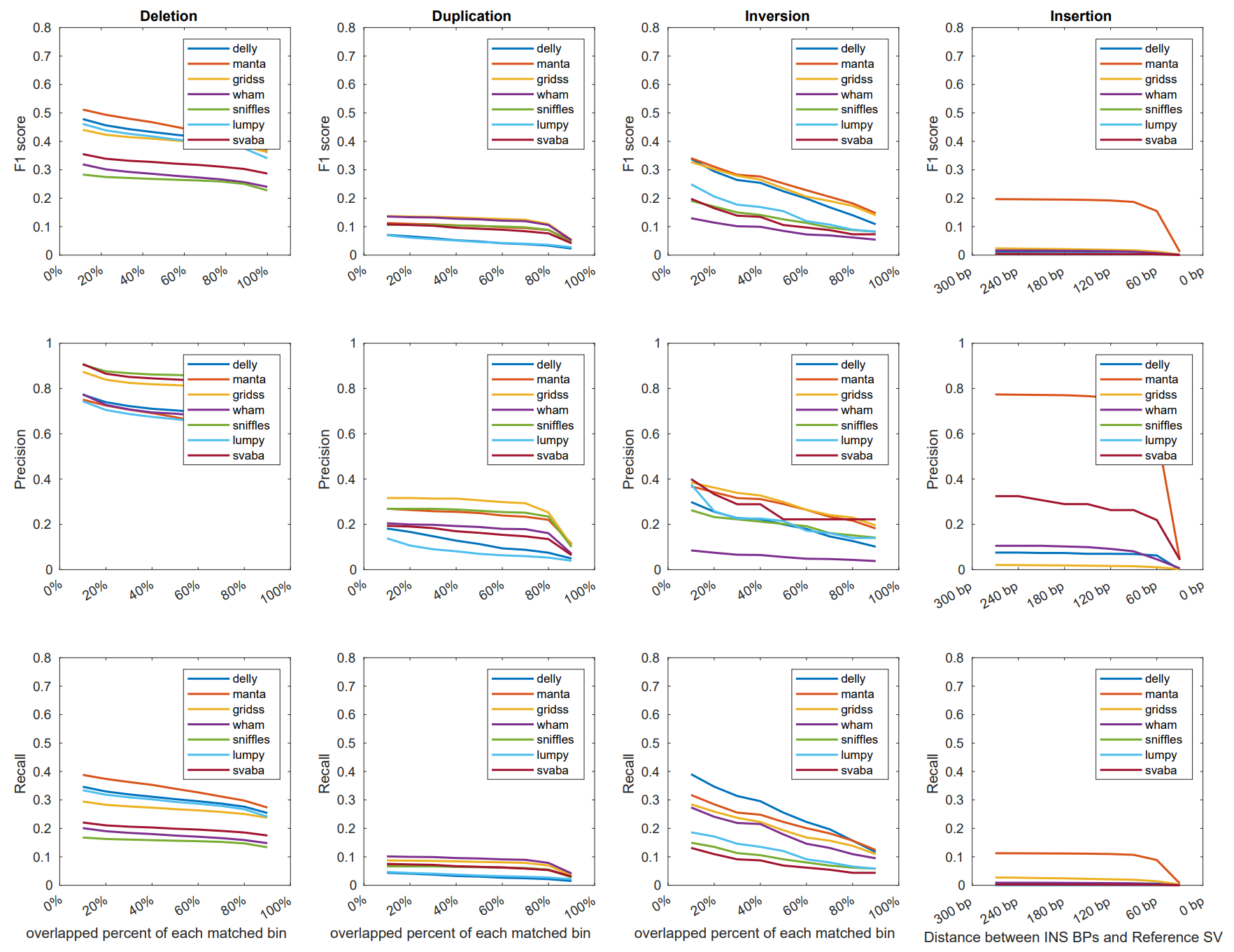
**

**S6 Fig.** **Structural variation (SV) detection performance of seven callers in 30x NA12878.**

**
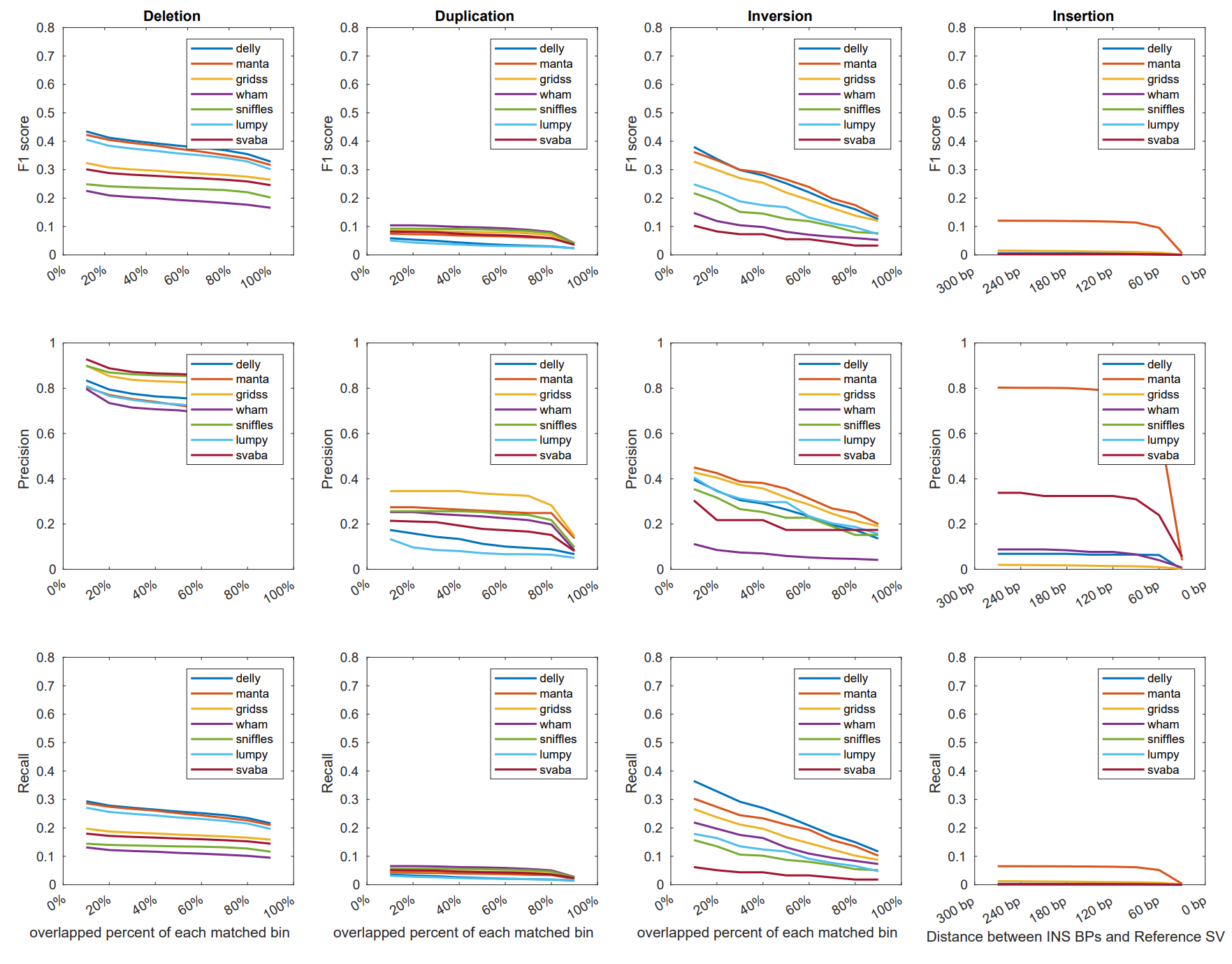
**

**S7 Fig.** **Structural variation (SV) detection performance of seven callers in 15x NA12878.**

**
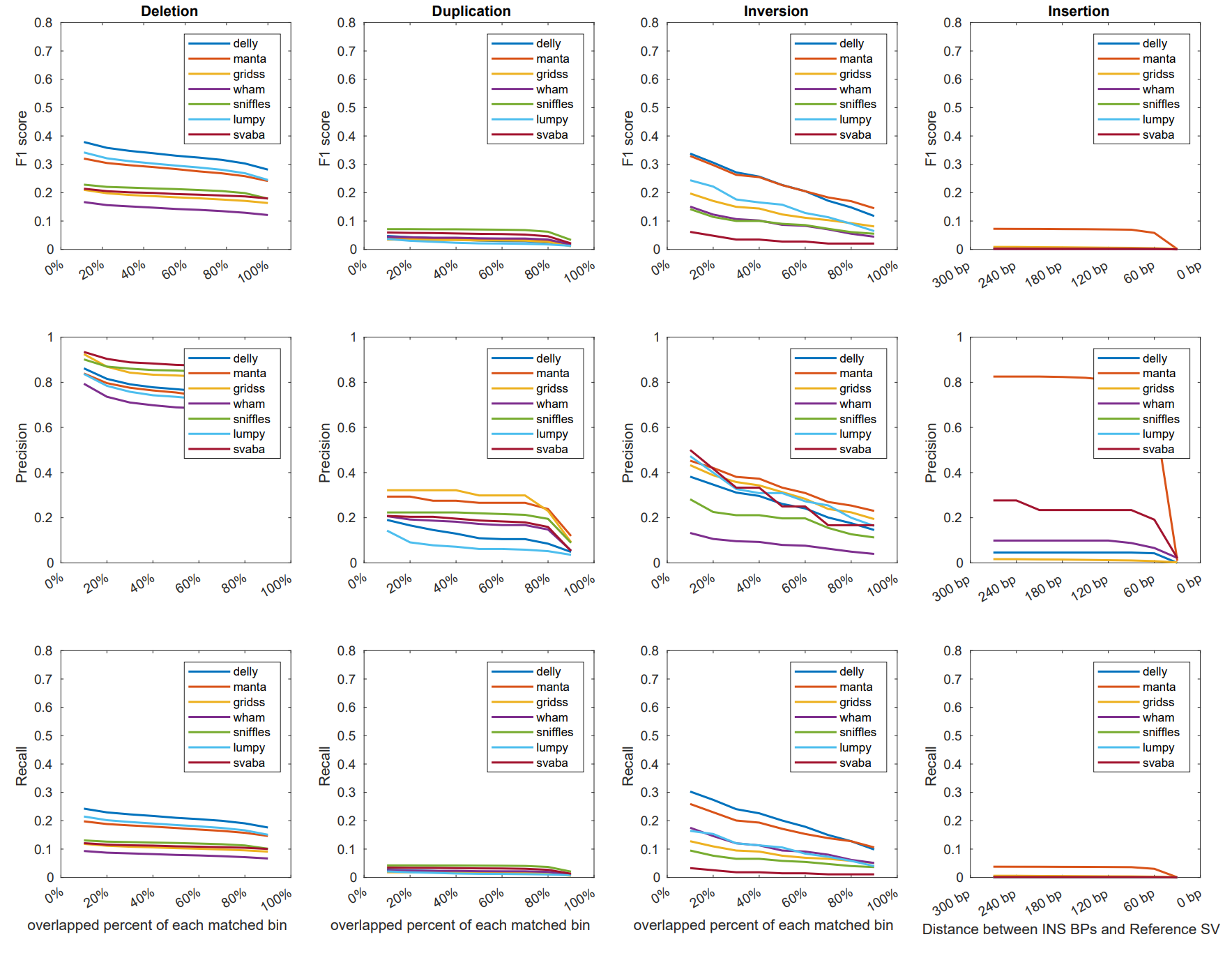
**

**S8 Fig.** **Structural variation (SV) detection performance of seven callers in 10x NA12878.**

**
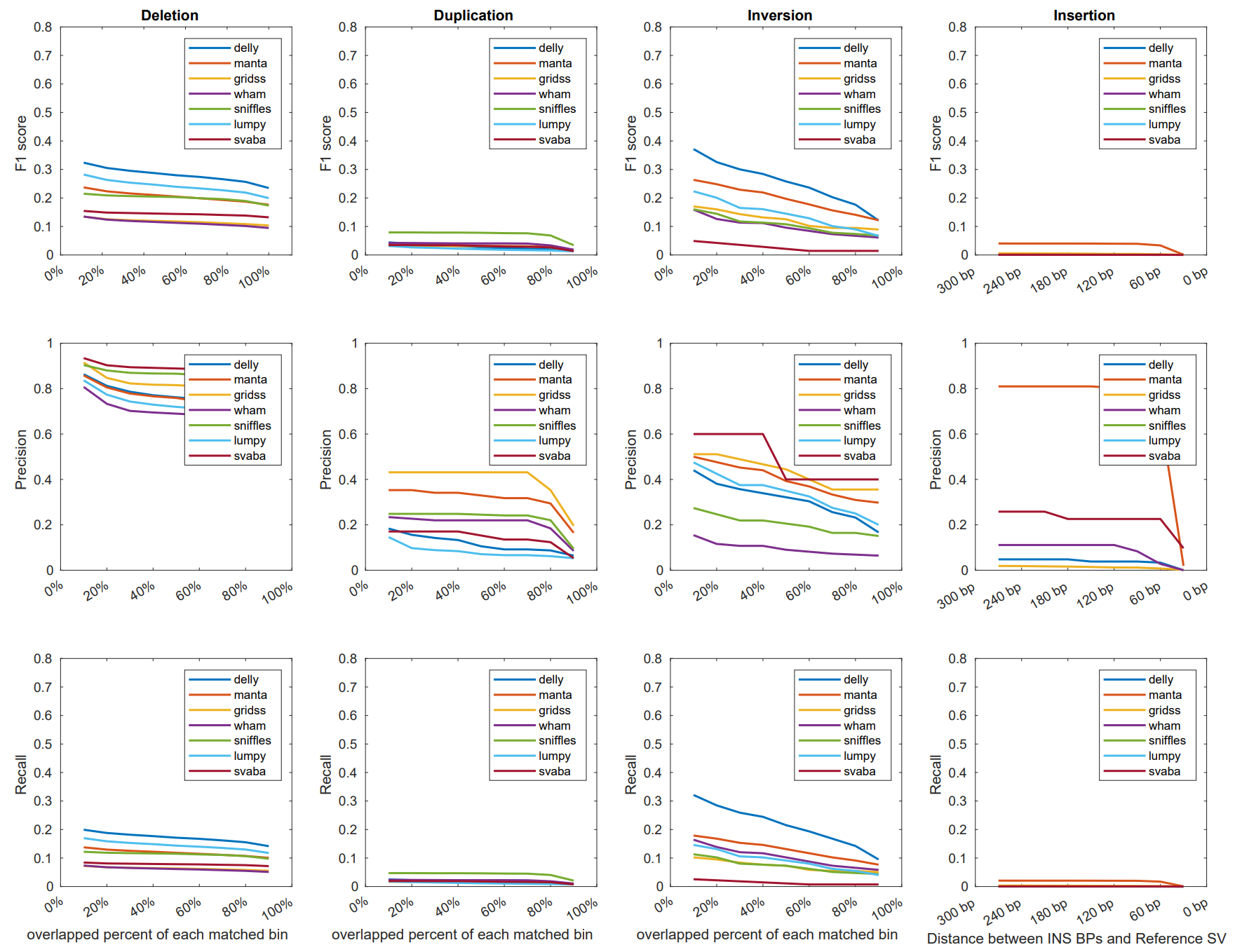
**

**S9 Fig.** **Structural variation (SV) detection performance of seven callers in 7x NA12878.**

**
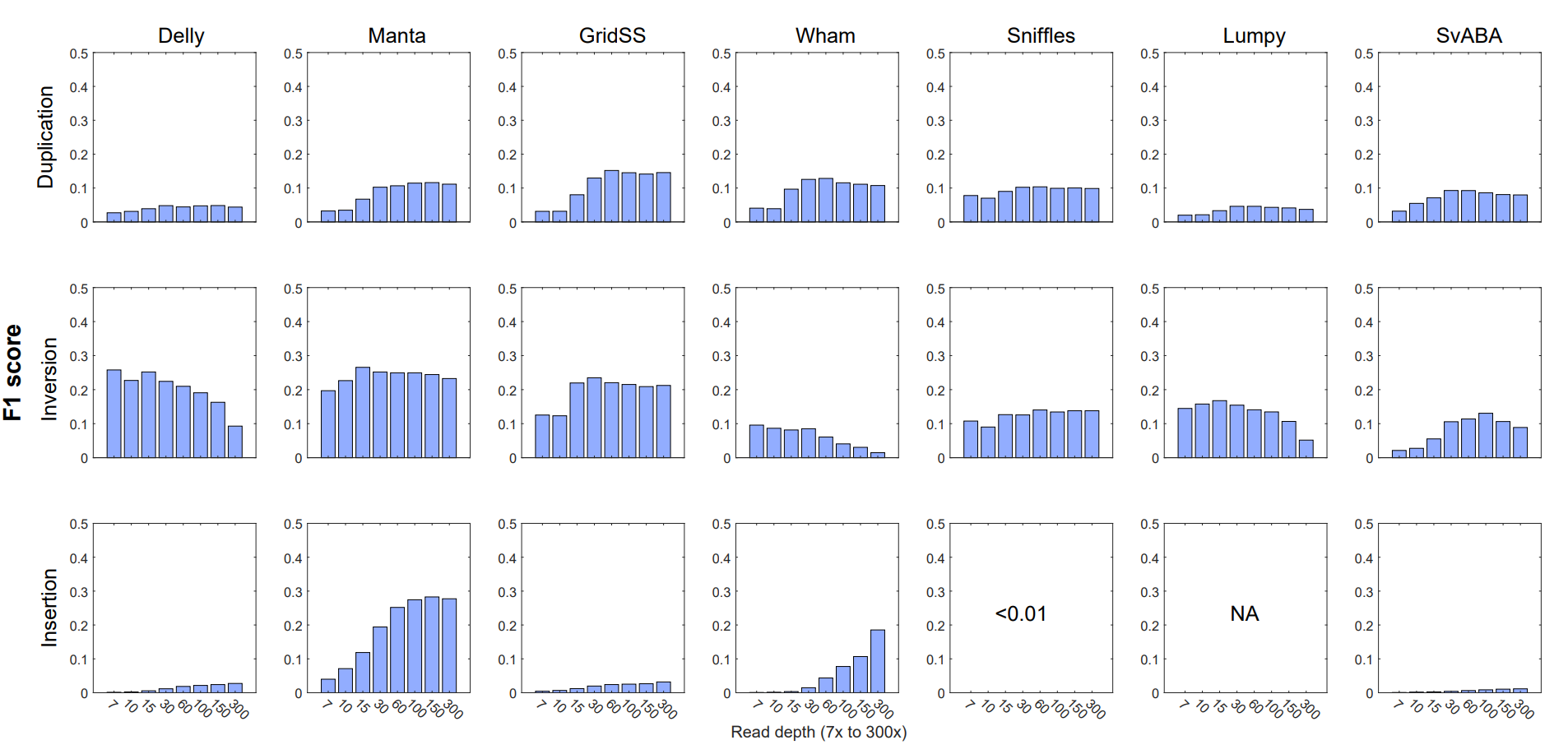
**

**S10 Fig.** **Structural variation (SV) detection performance according to read-depth. F1 scores of three SV types of seven SV callers according to read-depth.**

**
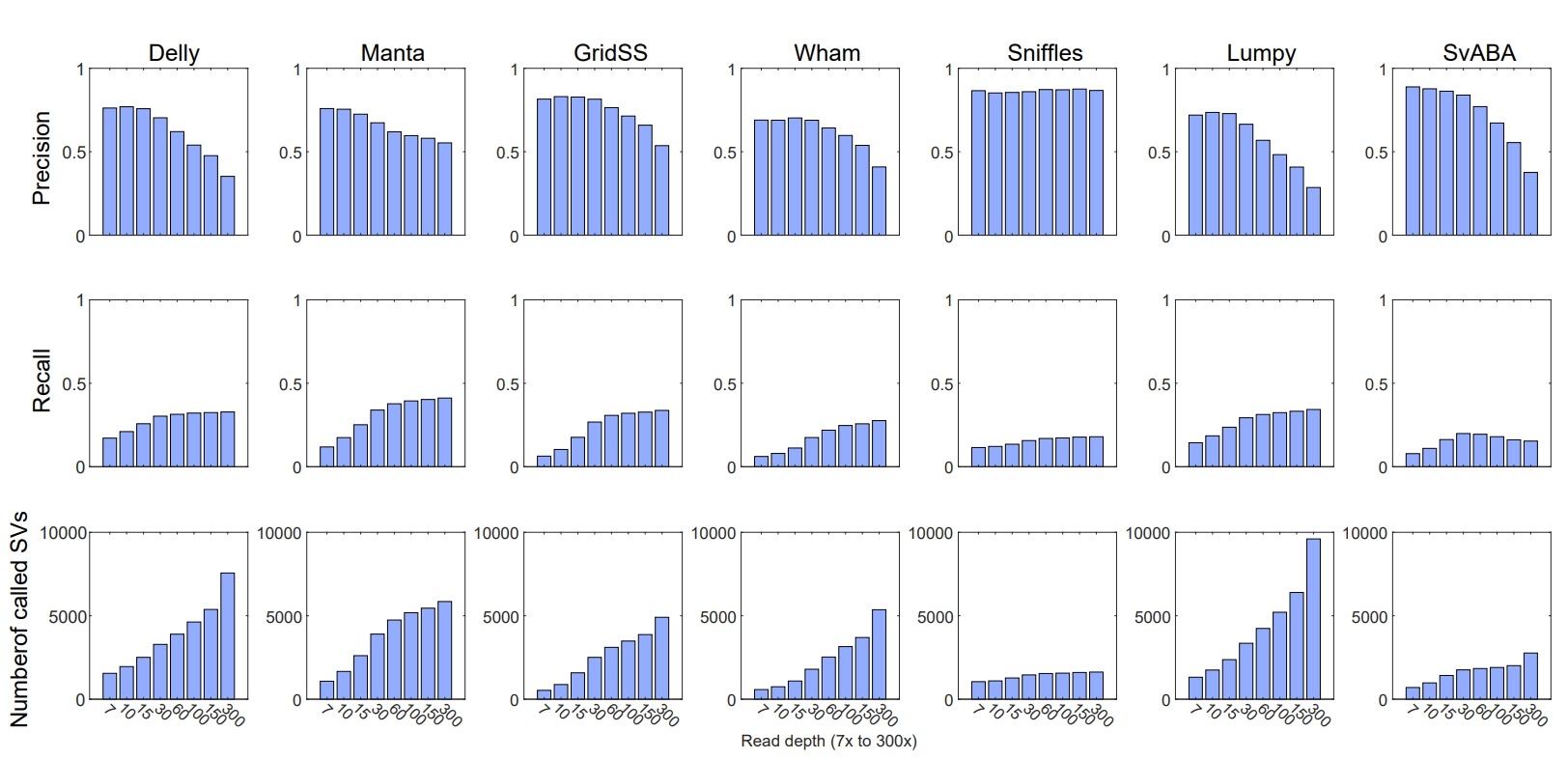
**

**S11 Fig.** **Structural variation (SV for deletion) detection performance according to read-depth.** Precision, recall, and number of detected deletions of seven SV callers according to read-depth.

**
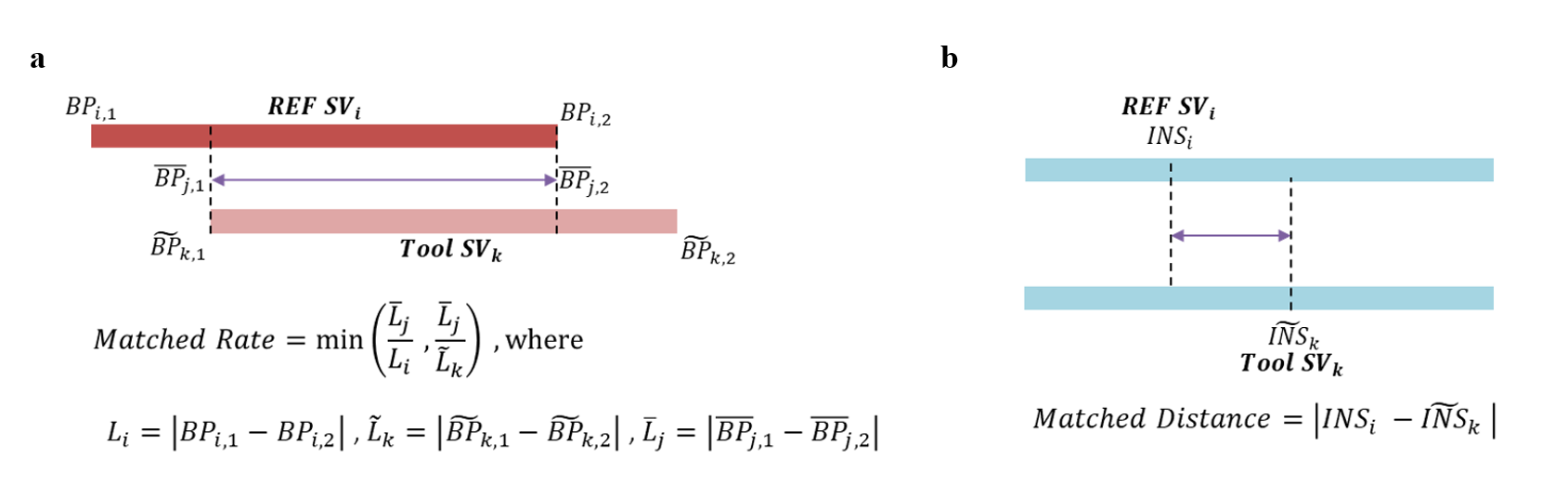
**

**S12 Fig.** **Estimation of structural variation (SV) correction between reference SVs and caller SVs.** To calculate the accuracy of SV detection in this study, we adapted the matching rule of SV pairs. a. For range SV, we used the matched rate explained in the a. b. For insertion, we used the absolute distance between the genomic locus of reference SVs and caller SVs. We used precision, recall, and F1 as evaluation metrics. For precision, we identified the ratio of true positive (TP) and sum of TP and false positive (FP) (i.e., precision=TP/(TP+FP)) of the predicted SVs from each caller. Here, TP and FP are calculated from the predicted SVs from each caller, and the matched ratio is calculated by a reciprocally overlapped range ratio. For recall, we identified the ratio of TP and sum of TP and false negative (FN) (i.e., recall=TP/(TP+FN) from reference SVs. Here, TP and FN are calculated from reference SVs, and the matched ratio is calculated by reciprocally overlapped range ratio. We also identified the F1 score as the harmonic mean of the precision and recall (i.e., F1=2ⅹ(precisionⅹrecall)/(precision + recall)).

**S1 Table. Whole genome sequence (WGS) dataset used in this study.**

| **Dataset** | **Samples** | **Sample ID** | **Data Repository** | **Read depth** | **Read length** | **Raw reads  (paired)** | **Trimmed reads  (paired)** | **Mapped reads** | **Insert size** | **Data repository URL** | **Reference** |
| --- | --- | --- | --- | --- | --- | --- | --- | --- | --- | --- | --- |
| General | HG00514 | ERR894729 | ERA | 25 | 126 | 256363602 | 206189712 | 409644513 | 559.9 | https://www.ncbi.nlm.nih.gov/sra | hg38 |
| General | HG00514 | ERR903030 | ERA | 25 | 126 | 263520907 | 216181674 | 429493424 | 559.3 | https://www.ncbi.nlm.nih.gov/sra | hg38 |
| General | NA12878 | SRR622457 | SRA | 85 | 101 | 1436823773 | 910319779 | 1639824722 | 392.8 | https://www.ncbi.nlm.nih.gov/sra | hg38 |
| 300x | NA12878 | NA12878-1 | *GIAB | 300 | 148 | 3099165624 | 2691496775 | 5270474582 | 538.9 | https://github.com/genome-in-a-bottle | hg38 |
| Downsampled | NA12878 | NA12878-2 | GIAB | 150 | 148 | NA | 1345748388 | 2650356852 | 538.8 | https://github.com/genome-in-a-bottle | hg38 |
| Downsampled | NA12878 | NA12878-3 | GIAB | 100 | 148 | NA | 897165592 | 1771414149 | 538.7 | https://github.com/genome-in-a-bottle | hg38 |
| Downsampled | NA12878 | NA12878-4 | GIAB | 60 | 148 | NA | 538299355 | 1065561862 | 538.7 | https://github.com/genome-in-a-bottle | hg38 |
| Downsampled | NA12878 | NA12878-5 | GIAB | 30 | 148 | NA | 269149678 | 534134244 | 538.6 | https://github.com/genome-in-a-bottle | hg38 |
| Downsampled | NA12878 | NA12878-6 | GIAB | 15 | 148 | NA | 134574839 | 267552416 | 538.4 | https://github.com/genome-in-a-bottle | hg38 |
| Downsampled | NA12878 | NA12878-7 | GIAB | 10 | 148 | NA | 89716559 | 178510915 | 538.3 | https://github.com/genome-in-a-bottle | hg38 |
| Downsampled | NA12878 | NA12878-8 | GIAB | 7 | 148 | NA | 67287419 | 133948473 | 538.2 | https://github.com/genome-in-a-bottle | hg38 |
| External | 601792 | K-001 | KoNA | 40 | 150 | 367707760 | 340526827 | 671289920 | 314.6 | **https://www.kobic.re.kr/kona/ | chm13 |
| External | 596346 | K-002 | KoNA | 40 | 150 | 452729923 | 423201894 | 837312202 | 315 | https://www.kobic.re.kr/kona/ | chm13 |
| External | 596730 | K-003 | KoNA | 40 | 150 | 407659135 | 371140610 | 736978497 | 313 | https://www.kobic.re.kr/kona/ | chm13 |

*GIAB (Genome in a Bottle): ftp://ftp-trace.ncbi.nih.gov/ReferenceSamples/giab/data/NA12878/NIST_NA12878_HG001_HiSeq_300x/
** The current link is reviewing page for available dataset for reviewers and editors

**S2 Table. Simulation data performances of structural variation (SV) callers.**

| Callers | **30x** | | | **60x** | | |
| --- | --- | --- | --- | --- | --- | --- |
|  | **Precision** | **Recall** | **F1** | **Precision** | **Recall** | **F1** |
| Delly | 0.958075 | 0.697634 | 0.807371 | 0.948602 | 0.739849 | 0.831321 |
| Manta | 0.983555 | 0.360312 | 0.527413 | 0.975967 | 0.502017 | 0.663 |
| GridSS | 0.990728 | 0.109169 | 0.196667 | 0.982972 | 0.430492 | 0.598758 |
| Wham | 0.845638 | 0.070987 | 0.130979 | 0.826633 | 0.225733 | 0.354626 |
| Sniffles | 0.96875 | 0.004437 | 0.008833 | 0.916667 | 0.003764 | 0.007498 |
| Lumpy | 0.963489 | 0.707717 | 0.816031 | 0.954144 | 0.794165 | 0.866835 |
| SvABA | 0.979977 | 0.979977 | 0.396236 | 0.967838 | 0.967838 | 0.58632 |

**S3 Table. Structural variation (SV) callers used in this study.**

| Callers | source code | version |
| --- | --- | --- |
| Delly | https://github.com/dellycallers/delly | v1.1.8 |
| Manta | https://github.com/Illumina/manta | v1.6.0 |
| GridSS | <https://github.com/PapenfussLab/GRIDSS> | v2.13.2 |
| Wham | https://github.com/zeeev/wham | v1.8.0 |
| Sniffles | https://github.com/fritzsedlazeck/Sniffles | v2.2 |
| Lumpy | https://github.com/arq5x/lumpy-sv | v0.3.1 |
| SvABA | https://github.com/walaj/svaba | v1.2.0 |
| Canvas | https://github.com/Illumina/canvas | v1.40.0.1613 |
| CNVnator | https://github.com/abyzovlab/CNVnator | v0.4.1 |
| MELT | https://melt.igs.umaryland.edu/downloads.php | v2.2.2 |
| INSurVeyor | https://github.com/kensung-lab/INSurVeyor | v1.1.2 |
